# Acetabular fracture and central hip dislocation in osteogenesis imperfecta child treated surgically with bone grafts augmented by recombinant human bone morphogenetic protein-2: A rare case report

**DOI:** 10.1016/j.ijscr.2023.108436

**Published:** 2023-06-22

**Authors:** Bintang Soetjahjo, Denny Adriansyah, Ahmad Fauzan, Benedictus Anindita Satmoko

**Affiliations:** a Orthopedic and Traumatology Department, dr. Moewardi General Hospital, Surakarta, Indonesia; b Faculty of Medicine, Sebelas Maret University, Surakarta, Indonesia; c Faculty of Medicine, Gadjah Mada University, Yogyakarta, Indonesia

**Keywords:** Osteogenesis imperfecta, Acetabular fracture, Central hip dislocation, Recombinant human bone morphogenetic-2, Open reduction internal fixation, Case report

## Abstract

**Introduction and importance:**

Rare congenital disorder osteogenesis imperfecta (OI) can make treating complex acetabular fractures-dislocations challenging. Open reduction and internal fixation (ORIF) with locking plates and screws may not produce satisfactory results. We present the outcome of ORIF with reconstruction locking plate and screw augmented by bone grafts with recombinant bone morphogenetic protein-2 (rhBMP-2) for OI type I child with Judet-Letournel both column type acetabular fracture associated with central hip dislocation.

**Case presentation:**

We present a case of a 13-year-old female OI type I patient with right hip pain after falling while biking. Both eyes had blue sclera and OI family history. Intraoperatively, the Stoppa approach was used. Proximal femoral skeletal traction was used to reduce the femoral head and aid bone graft reconstruction of the acetabular wall. Intraosseous injection of rhBMP-2 was added. Fractures were fixed with a curved reconstruction locking plate and screws. Bones and soft tissues were gently manipulated to prevent blood loss. Radiographic and functional results were remarkable.

**Clinical discussion:**

Fractures and blood loss are more likely to occur in OI type I patients due to collagen type I deficiency. Proximal femur skeletal traction is crucial for ORIF plating in acetabular fractures with central hip dislocation. This minimizes bone and soft tissue manipulation. RhBMP-2-injected bone grafts have structural support and osteoinductive properties that enhance bone healing. Despite its exceptional results in this case, further research is needed.

**Conclusion:**

The combination of our technique and rhBMP-2 effectively accelerates bone healing in OI patient treated with ORIF.

## Highlights

• Osteogenesis imperfecta (OI) may cause complicated fracture patterns.
• OI patient presents extra challenges in reducing and fixing fractures.
• rhBMP-2 acts as an osteoinduction factor and enhances bone healing.
• Bone healing showed exceptional results in this rare case report.

## 1 Introduction and importance

In osteogenesis imperfecta (OI), connective tissue production is impaired qualitatively and quantitatively, resulting in brittle bones, and is caused by an autosomal dominant defect in the collagen gene (COL1A1 or COL1A2) [1]. There is an extremely low incidence of OI, approximately one in every 20.000 births [2]. This heritable connective tissue disorder is characterized by low bone mass and increased fracture likelihood [3].

The acetabular fracture occurs after traumatic central hip dislocation due to axial loading on the femur during abduction [4]. The displaced fractures can be treated with open reduction and internal fixation (ORIF). This management can be challenging in the OI setting due to several factors, including bleeding diathesis, friable tissues, and possible deformities, including protrusion of the femoral head from the acetabulum and low bone density. Additionally, surgery reduces bone stock and increases fracture risk [5].

In activating the osteogenic pathway, bone morphogenetic protein-2 (BMP-2) promotes cell chemotaxis, proliferation, and differentiation of mesenchymal stem cells into bone and cartilage [6]. Currently, recombinant human BMP-2 (rhBMP-2) is made using recombinant human gene technology. Using intraosseous rhBMP-2 in OI mice increases periosteal bone formation, which may be beneficial in pediatric OI settings. Nevertheless, clinical implications and functional improvements have not yet been investigated [7]. This paper aims to present the outcome of ORIF with a reconstruction plate augmented by bone grafts with recombinant bone morphogenetic protein-2 for OI type I child with Judet-Letournel both columns type acetabular fracture associated with central hip dislocation. This case report has been reported in line with the Surgical Case Report (SCARE) 2020 Criteria [8].

## 2 Case presentation

A 13-year-old female student with right hip pain was admitted to our emergency department (height 140 cm; weight 35 kg; BMI 17.9 kg/m2). Six hours before admission, she fell from a slippery road while riding a bicycle, injuring her right hip. After the incident, she could stand and walk without aid (antalgic gait), but the pain in the right hip increased. Then, her family brought her to another peripheral hospital before being referred by ambulance to our hospital. At the prior hospital, an x-ray of the right hip was taken after an analgesic injection. No previous injuries or complaints history were obtained, such as nausea, vomiting, or fainting. A bone fracture or surgery had never occurred before. Her mother and sister both suffered from OI. The COVID-19 test was clear.

**Fig. 1:**
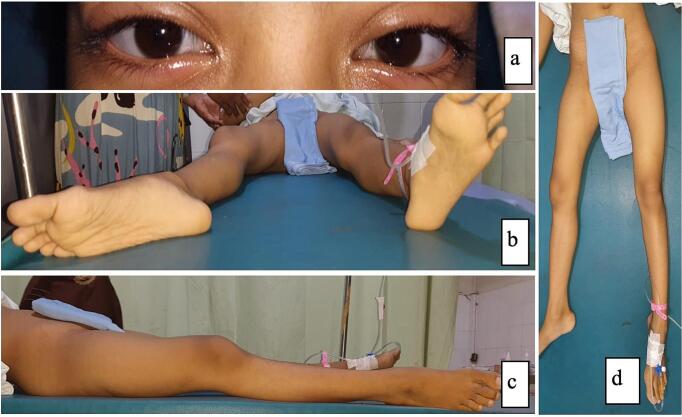
The clinical picture of the patient: **(a)** blue sclera, **(b)** abduction and external rotation of right leg position, **(c)** no bruising or swelling over the right hip, **(d)** shortening of the right lower limb.

Patent airway, spontaneous breathing, and stable hemodynamics were found during the primary survey. The blue sclera was observed in both eyes without dental malformations or short stature. Leg shortening with abduction and external rotation was evident on the corresponding lower limb. A 1 cm leg length discrepancy was shown in the apparent length (R: 77 cm / L: 78 cm) and the true length (R: 71 cm / L: 72 cm), but no anatomical length difference was observed. We observed a stable pelvic ring, no swelling, and bruising over the right hip, but we found tenderness around the right hip. The right hip joint range of motion (ROM) was limited (35° of flexion, 10° of abduction, 10° of external rotation, 0° of adduction) with a numeric pain rating scale (NPRS) of 6 and the Harris Hip Score (HHS) was 54.8 (poor). The clinical presentation of this patient is shown in Fig. 1.

(Source: internal documentation)

**Fig. 2:**
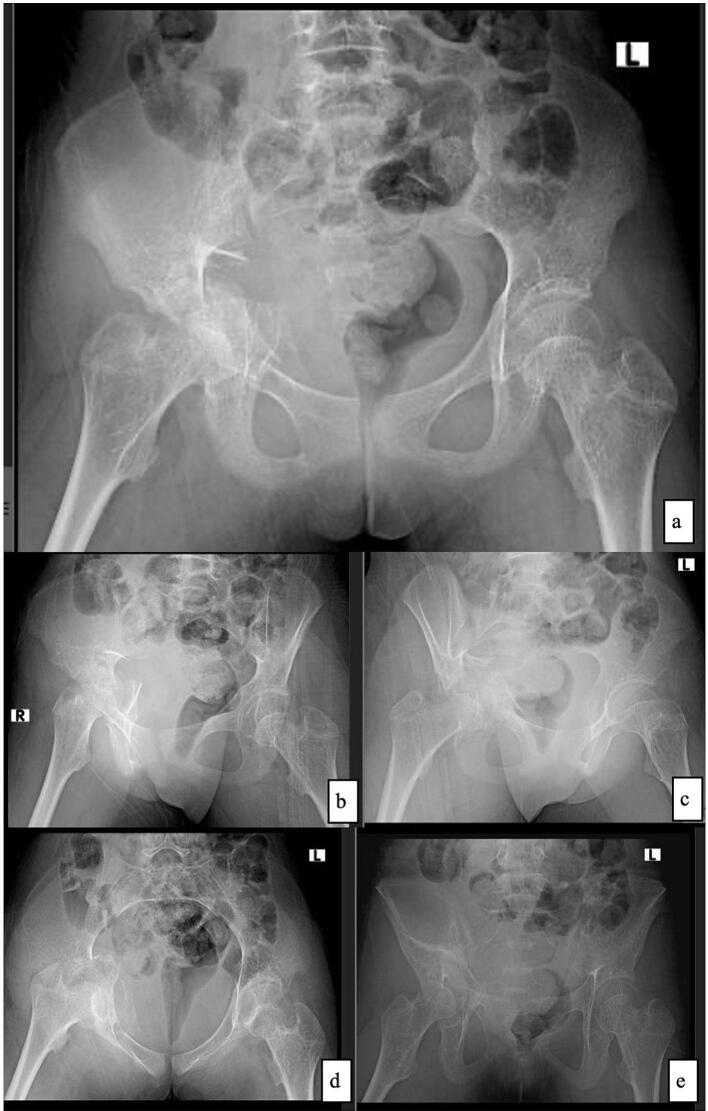
The pre-operative radiographic plain x-ray of the pelvis: **(a)** AP view, **(b)** right iliac oblique view, **(c)** right obturator oblique view, **(d)** pelvic inlet view, **(e)** pelvic outlet view showing the discontinuity of acetabular rim with comminuted fracture combined with central dislocation of the femoral head.

**Fig. 3:**
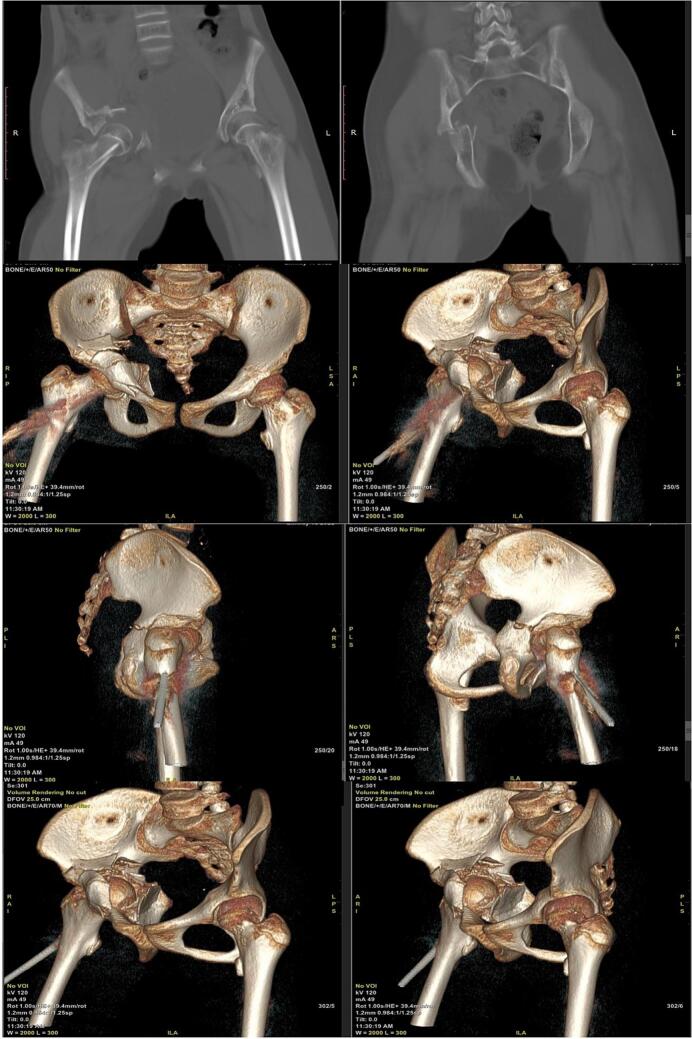
The reconstruction 3D-CT scan of the pelvis showing a severe degree of both column acetabular fracture with central hip dislocation.

The radiological examination of the X-ray pelvis was taken in anteroposterior (AP), right obturator oblique, right iliac oblique, pelvic inlet, and pelvic outlet views, as shown in Fig. 2. The bone density showed general rarefaction with discontinuity of the right acetabular rim and comminuted fracture associated with slight intrapelvic displacement of the femoral head. Neither the femoral head nor neck fractures complicated the acetabular fracture. Three-dimensional computed tomography (3D-CT) scans revealed greater comminution and displacement than plain radiographs (Fig. 3). In the right acetabulum, the anterior column showed a comminuted fracture, and the posterior column showed a slightly displaced transverse fracture. In this 3D-CT scan, the discontinuity of the acetabular rim that leads to femoral head dislocation is visible due to severe comminution of the anterior column acetabulum. This patient was diagnosed with OI type I with an acetabular fracture Judet-Letournel both of column type and central hip dislocation. We planned to do ORIF with a reconstruction plate and bone graft augmented by rhBMP-2.

(Source: internal documentation)

(Source: internal documentation)

## 3 Surgical procedure

The patient underwent elective surgery following two days of hospitalization to prepare for blood product cross-matching regarding potential complications associated with OI surgery. The patient was supine and under general anaesthesia, draped circumferentially around the right limb and anterior pelvis. A Schanz screw was percutaneously inserted into the proximal femur through the lateral greater trochanter under an image intensifier to reduce the femoral head. A Stoppa approach through the medial window of an ilioinguinal approach using a split of the musculus rectus abdominis was performed to access the fracture site. The femoral head was found to impact the acetabulum with a comminuted fracture of the anterior column and a transverse fracture of the posterior column. The centrally dislocated femoral head was reduced and maintained with skeletal traction before fixation. During the reduction of the femoral head, the posterior column was reduced by ligamentotaxis. Since the bones were fragile, we performed the anatomical reduction carefully. To fill the defect in the anterior column, we utilized five pieces of hydroxyapatite cancellous bone graft Bonefill® Ortho (Phapros Tbk, Indonesia) size 10 mm × 10 mm each. A nine-hole 3.5 mm curved locking reconstruction plate was shaped and placed along the fractured anterior column. The plate was fixed to the anterior column with eight cancellous locking screws. To prevent the femoral head from advancing, skeletal traction is maintained to aid bone healing. A 0.5 mg intraosseous injection of rhBMP-2 NOVOSIS™ (CGBio Co., Ltd., Korea) was injected into the bone graft around the anterior column fracture site to enhance bone healing.

**Fig. 4:**
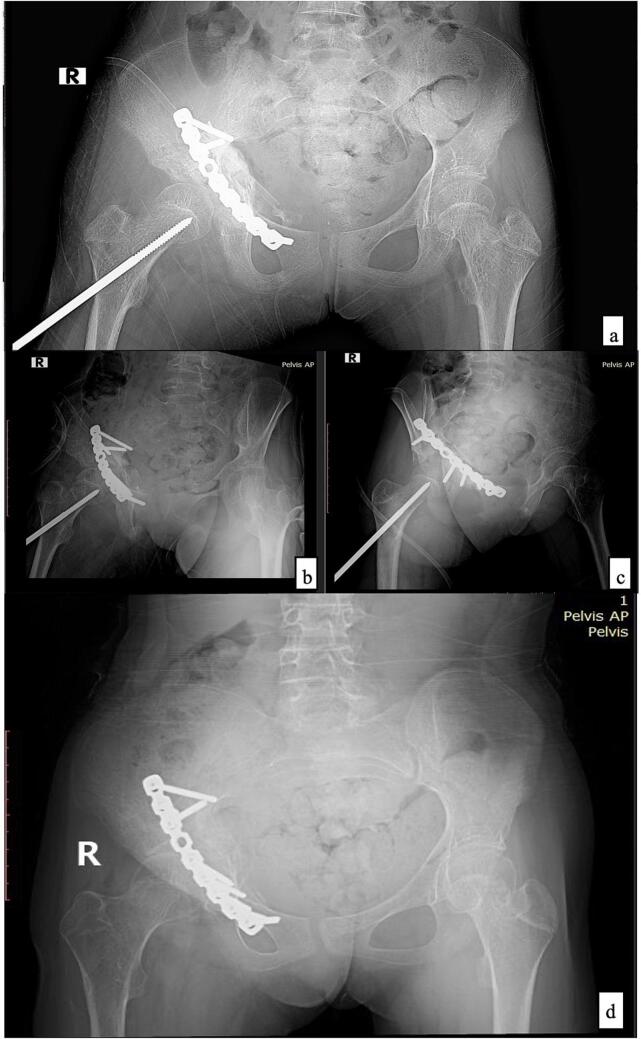
Post-operative radiological examination of pelvis X-ray: **(a)** AP view, **(b)** right iliac oblique view, **(c)** right obturator oblique view, **(d)** pelvis AP view follow-up evaluation post skeletal traction removal.

A drain was placed to accommodate post-operative bleeding before closing the incision and dressing the operation site. A blood transfusion was unnecessary since the intraoperative blood loss was less than 400 ml. Surgical complications were not observed. Our senior orthopedic surgeon, the first author, conducted this procedure in 1 h. Fig. 4(a-c) illustrates the post-operative pelvic X-ray in which both columns of acetabulum have been reduced and held by locking reconstruction plate and screw in the appropriate anatomical position and noticeable bone graft filling in the anterior column.

(Source: internal documentation)

Post-operative.

**Fig. 5:**
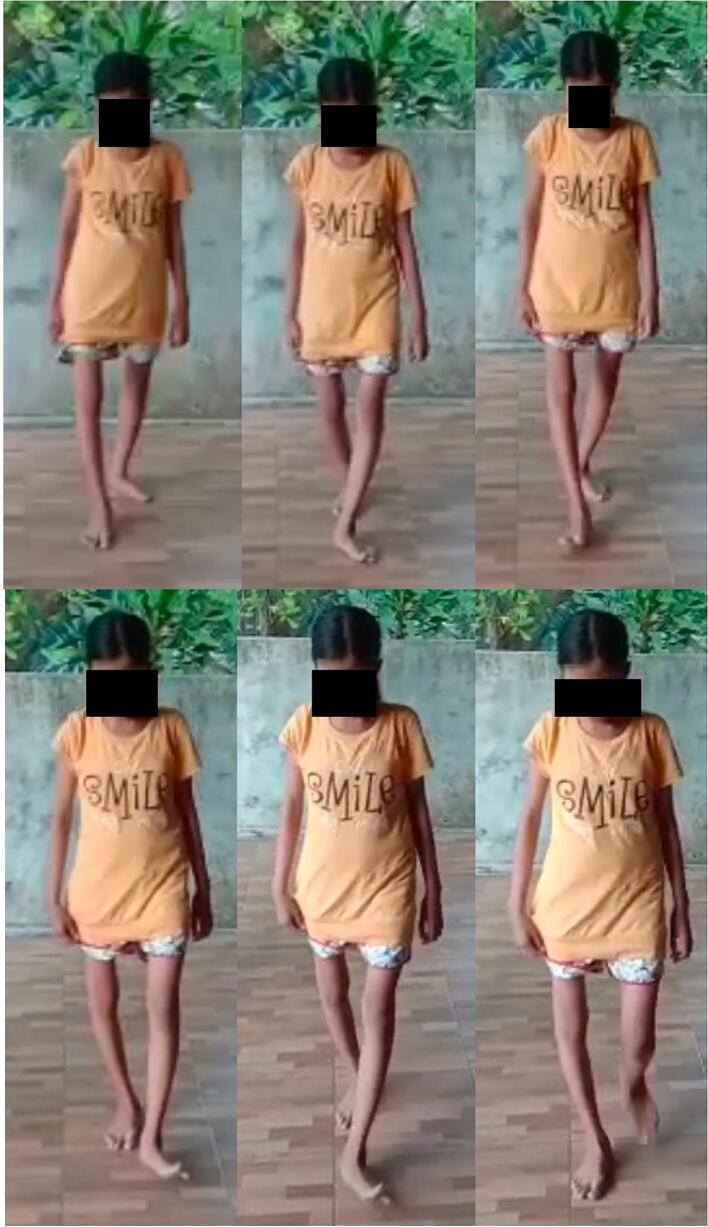
One-year clinical evaluation showed the patient can walk without aid.

After the post-operative pain was manageable, the active and passive range of motion exercises was performed with isotonic-isometric muscle contractions of the right hip joint. After a week of hospitalization, she was discharged with 3 mg/3 ml bisphosphonate injections every three months. We maintained skeletal traction for four weeks with loads between four and seven kilograms. Radiographs of four weeks after proximal femur skeletal traction removal showed adequate plate and screw fixation in the anatomical position of the anterior column acetabulum with adequate callus formation, as shown in Fig. 4(d). Rehabilitation continued with seated mobilization. The second month after surgery, partial weight bearing was permitted using crutches. Three months after surgery, full weight-bearing rehabilitation began. To measure the effectiveness of our intervention, we applied the NPRS, HHS and hip joint ROM as objective measurements. At four months follow-up, the NPRS and HHS were 2 and 95.65 (excellent), respectively. The right hip joint ROM showed 90° of flexion, 10° of abduction, 10° of external rotation, and 5° of adduction. The NPRS and HHS were 0 and 97 in a one-year evaluation, respectively. The right hip joint ROM showed 102° of flexion, 18° of abduction, 12° of external rotation, and 10° of adduction. Based on a clinical evaluation, she could resume her daily activity without a walking aid and only slight restriction of ROM in the right hip joint, as shown in Fig. 5. No hypertrophic callus or hypertrophic ossification (HO) was found.

(Source: internal documentation)

## 4 Clinical discussion

OI type I has a distinctive blue sclera and is the mildest non-deforming form [1,2]. OI patients also have an inherent coagulation defect caused by abnormal collagen within endothelial capillaries. Therefore, when choosing an operative decision, we should consider minimal soft tissue injury and bone manipulation because bleeding diathesis is expected in 10 % to 30 % of OI patients, besides intraoperative fracture and poor bone stock [9].

High-energy trauma can cause hip fracture-dislocations involving acetabular fractures, which need immediate reduction and surgery to restore joint stability. There may be multiple surgical approaches depending on the fracture/dislocation pattern. Thus, the procedure will take longer surgical time, more blood loss, and complications such as avascular necrosis of the femoral head, osteoarthritis, and HO [10,11]. Fractures in OI patients typically occur in young patients who have been traumatized with low energy [12]. Despite recent locking plates, there is still a higher risk of malunion and non-union, poor callus quality, and fixation destabilization because of low bony support [13]. Moreover, plate fixation is associated with high peri-implant fracture risks in OI patients.

The effects of excessive immobilization on non-operative management can be detrimental since it leads to weak, stiff muscles, osteopenia, and fractures [14]. Precise surgical technique combined with a meticulous preoperative plan can achieve a satisfactory clinical and functional result [15]. A 5-year follow-up reported by Zawadski et al. on a geriatric OI type I patient treated with ORIF and a reconstruction plate for pelvic ring fracture showed superior clinical and radiographic results [12]. Meanwhile, Medici et al. published a case of bilateral acetabular fracture with protrusio acetabulum in an OI type I patient treated in 2 stages. Although the results are unacceptable, the main objective is to delay a joint replacement as long as possible due to the young patient [5].

BMP-2, a bone anabolic protein, can promote bone regeneration when delivered locally and may provide an alternative to bone grafting. Local injection of rhBMP2 could accelerate normal bone healing at the fracture site [16,17]. Luangphakdy et al. found that rhBMP-2 was effective in bone graft applications when applied to type I bovine collagen sponges [18]. According to Kim et al., rhBMP-2-soaked hydroxyapatite can be used in ridge augmentation to complement implant placement in edentulous regions [19]. In a systematic review, rhBMP-2 has been shown to be an effective alternative to the autogenous bone for the regeneration of height and width of bone so that a prosthesis can be supported by an implant and be stable for at least five years [20].

In this case, blue sclera in both eyes and family history supported OI type I diagnosis. A low-energy mechanism of injury caused acetabular fracture and central hip dislocation in this patient, suggesting that OI had compromised bone density. Due to friable tissue, poor bone quality, and a complex fracture pattern, surgical management strategies would be challenging. Considering the potential for prolonged immobilization and lowering bone mass, non-operative management may not be an option in this patient. Moreover, the joint replacement decision should be delayed due to the patient's age. Reconstruction of the acetabular wall in its anatomical position was crucial during traction. A locking plate and screws were used in cases of low bone mass to provide adequate fixation for bone healing. This procedure required minimal manipulation of surrounding tissue and bone. Because Medici et al. [5] reported an unsatisfactory result from ORIF with locking plates and screws in OI patients with acetabular fracture; we used rhBMP-2 in hydroxyapatite bone graft to accelerate bone healing instead of providing structural support at the fracture site. The skeletal traction should be maintained post-operatively to prevent recurring central dislocation and maintain the reduction to allow enough space for bone healing. This post-operative skeletal traction was recommended to the patient because the patient has fragile bones that could lead to loss of correction when the pressure/force is applied to the fixated fracture site. Thus, all methods were used for optimizing OI surgical treatment using the ORIF technique. As a result, minimal intraoperative blood loss was observed, and exceptional functional results were achieved. The improvement of the measurement scores within four months and as per the last evaluation showed the effectiveness of our methods. This report has several limitations, such as: (1) Long-term follow-up needs to be evaluated, (2) We only report a single patient, and (3) The clinical picture of the surgical procedure and process cannot be shown. Nevertheless, we consider this study essential to influence future studies of well-designed trials with larger sample sizes.

## 5 Conclusion

A minor injury may cause acetabular fractures with central hip dislocation in OI. Treating fracture dislocations with such complexity can be challenging. Preserving the original joint is mandatory to delay prosthetic replacements in young patients. Intraosseous rhBMP-2 injections may increase periosteal bone formation in pediatric OI. Although the ORIF locking plate and screw augmented with bone graft yielded exceptional results radiographically and functionally in this case report, long-term outcomes must be determined to compare this procedure with other options. Furthermore, well-designed studies are needed to provide alternative treatment options for OI patients with fracture-dislocations.

## Patient consent

Written informed consent was obtained from the patient for publication of this case report and accompanying images. A copy of the written consent is available for review by the Editor-in-Chief of this journal on request.

## Patient perspective

I was devastated by the fact that I would not be able to attend a school or perform my daily activities. Thanks to the physician's surgical technique and rehabilitation program, I could fulfil my daily activities, particularly schoolwork.

## Ethical approval

Ethical approval for this study (Ethical Clearance number 738/XI/HREC/2022) was provided by Health Research Ethics Committee of dr. Moewardi General Hospital, Surakarta, Indonesia on 09 November 2022.

A copy of ethical clearance letter is available for review by the Editor-in-Chief of this journal on request.

Bonefill® Ortho (Phapros Tbk, Indonesia), a resorbable calcium salt bone void filler device, was registered in Health Ministry of Indonesia, Directorate General of Pharmaceuticals and Medical Devices with distribution number KEMENKES RI AKD 21302910196 on March 2019.

NOVOSISTM (CGBio Co., Ltd., Korea), a resorbable calcium salt bone void filler device, was registered in Health Ministry of Indonesia, Directorate General of Pharmaceuticals and Medical Devices with distribution number KEMENKES RI AKL 21302815005 on September 2022.

## Funding

This case report received no specific grant from any funding agency in the public, or non-profit sector.

## Author contribution

Bintang Soetjahjo involved in conceptualization, case presentation, data collection, elaborating the surgical technique, main guidance for write up the manuscript.

Denny Adriansyah involved in conceptualization, case presentation, data collection, writing, reviewing, and editing the manuscript.

Ahmad Fauzan involved in data collection, conceptualization, reviewing, and editing the manuscript.

Benedictus Anindita Satmoko involved in conceptualization, writing, reviewing, and editing the manuscript.

## Guarantor

Bintang Soetjahjo; Orthopedic and Traumatology Department, Faculty of Medicine, Sebelas Maret/Dr. Moewardi General Hospital, Surakarta, Indonesia; Email: bjortho@yahoo.com

## Conflict of interest statement

The authors have no conflicts of interest to disclose.
